# Convergent Transcription in the Butyrolactone Regulon in *Streptomyces coelicolor* Confers a Bistable Genetic Switch for Antibiotic Biosynthesis

**DOI:** 10.1371/journal.pone.0021974

**Published:** 2011-07-12

**Authors:** Anushree Chatterjee, Laurie Drews, Sarika Mehra, Eriko Takano, Yiannis N. Kaznessis, Wei-Shou Hu

**Affiliations:** 1 Department of Chemical Engineering and Materials Science, University of Minnesota, Minneapolis, Minnesota, United States of America; 2 Department of Chemical Engineering, Indian Institute of Technology Bombay, Powai, Mumbai, India; 3 Department of Microbial Physiology, University of Groningen, Groningen, The Netherlands; University of Minnesota, United States of America

## Abstract

*cis*-encoded antisense RNAs (*cis* asRNA) have been reported to participate in gene expression regulation in both eukaryotic and prokaryotic organisms. Its presence in *Streptomyces coelicolor* has also been reported recently; however, its role has yet to be fully investigated. Using mathematical modeling we explore the role of *cis* asRNA produced as a result of convergent transcription in *scbA-scbR* genetic switch. *scbA* and *scbR* gene pair, encoding repressor–amplifier proteins respectively, mediates the synthesis of a signaling molecule, the γ-butyrolactone SCB1 and controls the onset of antibiotic production. Our model considers that transcriptional interference caused by convergent transcription of two opposing RNA polymerases results in fatal collision and transcriptional termination, which suppresses transcription efficiency. Additionally, convergent transcription causes sense and antisense interactions between complementary sequences from opposing strands, rendering the full length transcript inaccessible for translation. We evaluated the role of transcriptional interference and the antisense effect conferred by convergent transcription on the behavior of *scbA-scbR* system. Stability analysis showed that while transcriptional interference affects the system, it is asRNA that confers *scbA-scbR* system the characteristics of a bistable switch in response to the signaling molecule SCB1. With its critical role of regulating the onset of antibiotic synthesis the bistable behavior offers this two gene system the needed robustness to be a genetic switch. The convergent two gene system with potential of transcriptional interference is a frequent feature in various genomes. The possibility of asRNA regulation in other such gene-pairs is yet to be examined.

## Introduction

Transcription from a pair of promoters arranged in face-to-face orientation is ubiquitous both in bacteria and eukaryotes. It leads to a complete or partial overlap between convergent or divergent transcripts. Widespread convergent transcription leads to a large number of *cis* asRNA in eukaryotic genomes including human [1], mouse [2], *Drosophila* [3], *A. thaliana* [4], and yeast [5]. Many of these *cis* asRNA's are non-coding, but have been shown to participate in regulation [2], [6], [7]. Recent genomic analysis in bacteria has revealed a plethora of *cis*-encoded non-coding RNA in many species, including *E. coli* [8], *B. subtilis* [9], and *Mycobacterium tuberculosis* [10]. The regulatory role of convergent transcription in key biological decision making has been shown in a number of studies including, *prgQ-prgX* gene-pair in *E. faecalis* controlling transfer of plasmid pCF10 via conjugation [11], the *furA-alr1690 mRNA* in cyanobacterium *Anabena* sp. PCC 7120 regulating of Ferric uptake during environmental stress response [12] and the *mgtCBR-AmgR* locus in *S. enterica* controlling virulence in mice [13]. The discovery of *cis* asRNA in the model streptomycete *Streptomyces coelicolor* was reported only recently [14], [15], [16]. Despite the increasing evidence of antisense transcription, the regulatory role of convergent transcription has been not been investigated in *S. coelicolor*. In this work, the role of convergent transcription in the *scbA-scbR* system is evaluated.

The soil dwelling organism, *S. coelicolor* uses its arsenal of antibiotics to compete with other organisms in the environment. Its production of antibiotics is regulated by the synthesis of γ-butyrolactones, members of the quorum sensing-type family of signaling molecules [17], [18], which are found in many *Streptomyces* species, including *Streptomyces virginiae* [19], *Streptomcyes lavendulae* [20], [21], and *Streptomyces clavuligerus* [22]. In *S. coelicolor* A3(2), three kinds of γ-butyrolactones have been identified which serve to synchronize the onset of antibiotic synthesis within the population [23], [24], [25], among these *S. coelicolor* butanolide 1 (SCB1) is abundantly found. These SCBs regulate antibiotic biosynthetic clusters controlling synthesis of blue pigmented actinorhodin (Act) [26], red pigmented undecylprodigiosin (Red) [26] and yellow pigment yPCK [27].

**Figure 1:**
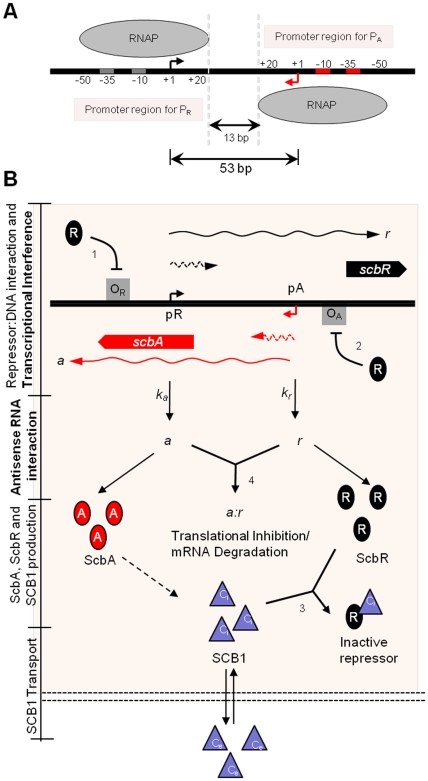
Convergent transcription in the *scbA-scbR* gene regulatory network. (**A**) Schematic of the promoter regions for pA and pR, and RNAP footprint at the respective promoters. (**B**) The *scbA-scbR* gene regulatory network. Convergent promoter pA-pR drive expression of genes *scbA* (shown in red) and *scbR* (shown in black) present on the opposite DNA strands (shown by black lines) to give rise to full-length transcripts *a* and *r* (RNA denoted by curved lines) and short truncated RNA (denoted by dashed-curved lines) respectively. **Transcriptional Interference model:** Collision between elongating RNAPs and between an elongating RNAP and a stationary RNAP at the opposing promoter causes transcriptional termination and results in the generation of short truncated transcripts (dashed-curved lines) from promoters pA and pR respectively. Full-length transcripts *a* and *r* result when elongating RNAPs escape collision. **Antisense Regulation:** Hybrid RNA complexes formed between full-length *a* and *r* RNA result in translational inhibition or mRNA degradation. Protein ScbR (R) can repress transcription from pA and pR (indicated by blunt arrows) by binding to operator sites O_A_ and O_R_ adjacent to promoters pA and pR respectively. Only full-length transcripts *a* and *r* are translated to protein ScbA (A) and ScbR (R). The intracellular γ-butyrolactone SCB1 (denoted by C_i_) is produced from glycerol derivatives and β-keto acid by the enzymatic action of γ-butyrolactone synthase ScbA. SCB1 forms complex with ScbR (C_i_R) to sequester its repressive effect. SCB1 diffuses out of the cell in to the extracellular environment and vice versa (denoted by C_e_). The reactions are numbered according to equations in Table 1.

The onset of antibiotic production has to be a tightly regulated process as antibiotics can be lethal even to their producers. The genetic switch controlling the transition from a non-producing state to an antibiotic producing state must be robust. Previously, it has been shown that *S. coelicolor* changes from a vegetative growth state without antibiotic production (OFF state) to an antibiotic producing state (ON state), upon induction with SCB1 [26]. This leads to amplification of the γ-butyrolactone SCB1 signal, resulting in a switch-like transition [26], [28]. A gene pair, *scbA* (SCO6266) and *scbR* (SCO6265), convergently transcribed from a set of face to face promoters, pA and pR respectively, regulates the biosynthesis of SCB1(Fig. 1) [26]. The s*cbR* gene encodes for a cytoplasmic receptor protein, ScbR (R), which has a γ-butyrolactone binding domain at its C-terminal and a DNA binding domain in its N-terminal [29], [30]. In absence of SCB1, ScbR auto-represses itself and represses *scbA* through binding at the O_R_ and O_A_ operator sites (Fig. 1A–B), flanking promoter pR and pA respectively [17], [26]. Additionally, in absence of signaling molecules, ScbR represses expression from the cryptic type I polyketide synthase gene cluster (*cpk*) by directly binding to promoter region of *cpkO*, the activator of the *cpk* gene cluster [31]. The *scbA* gene encodes for ScbA, a AfSA homologue, a key enzyme in synthesis of A-factor, the γ-butyrolactones in *S.gresius* [32]. ScbA has homology to fatty acid synthases and has been shown to be involved in the production of SCB1 from glycerol derivatives and β-keto acid derivatives as precursors [33]. At high concentrations, SCB1 binds to ScbR to form a SCB1-ScbR (CR) complex, thereby relieving its self-repression [26].

The proposed regulatory mechanism of the system was previously analyzed using a mathematical model and was shown to exhibit a bistable response of regulatory repressor ScbR levels at varying concentrations of the signaling molecule SCB1 [28]. In the model, ScbA and ScbR were postulated to form a protein complex (ScbA-ScbR) which acts as a positive regulator of ScbA [28], thus upregulating SCB1 synthesis. ScbA-ScbR formation was a key component contributing to the bistability. However, lack of experimental evidence for the ScbA-ScbR complex [28] prompted us to look for alternative mechanisms that could confer bistability to this system.

The *scbA* and *scbR* genes overlap by 53 bp from their respective transcription start sites [26] (Fig. 1A), resulting in a possible head-to-head collision of converging RNA polymerases (RNAPs) either between both elongating RNAPs or between an elongating RNAP and RNAP stationed at the opposing promoter serving as a sitting duck for collision. Such a suppressive influence of transcriptional activity of nearby or overlapping genes *in cis* is referred to as transcriptional interference (TI) [34], [35], [36], [37]. TI caused by RNAP collision leads to transcriptional termination, which results in decrease in expression of full-length RNA from promoters pA and pR and generation of truncated RNA (Fig. 1B) [30]. In addition to TI, the convergent transcription also generates transcripts that have a segment of complementary sequence, which may incur antisense interactions between sense-antisense full-length *scbR* (*r*) and *scbA* (*a*) transcripts resulting in translational inhibition or mRNA degradation of hybrid RNA complexes [38] (Fig. 1B). Here we show that convergent transcription from the *scbA-scbR* locus alone, without positive feedback from a hypothetical ScbA-ScbR protein complex, yields a robust bistable genetic switch in response to the signaling molecule SCB1. Similar switches could potentially operate in other two-gene systems arranged in convergent orientation in *S. coelicolor*.

## Results

The convergent transcription from pA and pR and the overlapping region of *scbA-scbR* is shown in Fig. 1A. The success or failure of each transcription initiation hinges on whether RNAP fires from or binds at promoter pA during the time taken by RNAP from pR to traverse the overlapping DNA and vice versa [39]. Our model incorporates three mechanisms of transcriptional interference, namely, (i) promoter occlusion, in which a passing RNAP originating at pR blocks access to pA and vice versa, (ii) collision between converging elongating RNAP originating from pA and pR and (iii) sitting duck collisions, in which closed promoter complex at pA is removed by collision with a passing RNAP originating at pR and vice versa [34], [40]. The footprint of an RNAP bound to the promoter is considered to extend between −50 to +20 bp [40], [41]. In an event where both pA and pR promoters are bound by RNAP, each RNAP can travel a maximum of 13 bp before a collision occurs (Fig. 1A). Collision between RNAPs regardless of whether both are elongating or one is bound to promoter is considered to be fatal for both the RNA polymerases [40]. It is assumed that the 3′ end-most base of a nascent transcript is 20 bp from the locus of the front end of RNAP (RNAP footprint), and this is used to calculate the length of a truncated RNA due to aborted transcription [40]. Binding of RNAP at one promoter is prevented (occlusion) once an elongating RNAP originating from the other reaches within 20 bp from the start site of the opposing promoter. With conditions stated above the maximum length of the truncated transcript resulting from aborted transcription would be 13 nt (Fig. 1A–B).

**Figure 2:**
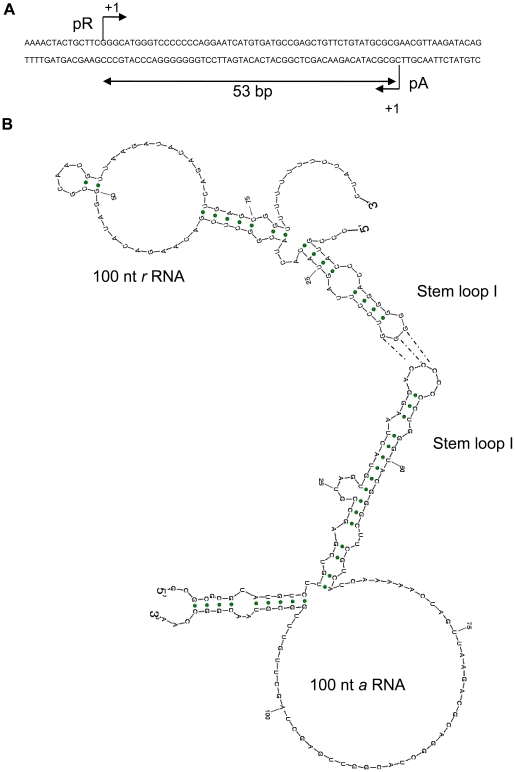
Antisense RNA within *scbA-scbR* overlapping locus. (**A**) Overlapping DNA sequence of *scbR-scbA* locus. (**B**) *In silico* RNA secondary structures of 100 nt pR and pA transcript representing the structures of full-length *r* and *a* RNA respectively. Stem loop I present on both nascent *a* and *r* RNA contain complementary single stranded regions.

The secondary structure of transcript in the overlapping region (Fig. 2A) was predicted using Sfold (Fig. 2B). The 100 nt pR transcript comprises of G-rich single-stranded region, indicated as Stem loop I in Fig. 2B. This stem loop is complementary to the RBS of 100 nt pA transcript [26], indicating likely sense–antisense RNA interaction with *scbA*. This is based on the fact that RNA interactions are more-likely to be mediated through stem-loop structures [42], [43], though presence of additional single-stranded regions complementary between *a* and *r* RNA could further enhance the potential of sense–antisense interactions between transcripts from this locus [42]. With the truncated RNA having a maximum size of 13 nt from transcription initiation site, and the stem loop region capable of sense-antisense interaction being located between 14–17 nt of pR transcript and 38–43 nt of pA transcript, we conclude that the sense-antisense interaction between the 13 nt truncated transcripts from pA and pR have weak interactions with the corresponding counterpart full-length transcripts; however, the interactions between full-length mRNAs from pA and pR are significant.

### Mathematical model of convergent transcription in the *scbA-scbR* locus

A mathematical model for the revised *scbA/scbR* system consisting of a set of 7 differential equations was formulated to describe the model shown in Fig. 1B. The binding and hybridization reactions, the equilibrium relationship and the mass action equations are shown in Tables 1 and 2 (Equations 1–15). The binding interactions between the ScbR repressor protein (R) and the operators O_R_ and O_A_ (Equations 1, 2), binding of ScbR to SCB1 (C) (Equation 3) are considered. Importantly, the model also considers antisense interaction between full-length transcripts (*a* and *r*) to result in formation of RNA hybrid complexes *a*:*r* (Equations 4). We assume that the interaction between truncated RNA (13 nt and shorter) and full length transcript can be neglected. Assuming binding of R to operators O_R_ and O_A_ reaches rapid equilibrium, the fraction of the unbound operator sites is given by Equations 5 and 6 respectively [28]. The synthesis rate of SCB1 is assumed to be proportional to the ScbA concentration and is given by the first term in Equation 13 [28].

**Table 1:** Reactions in *scbA-scbR* gene network.

Equation No.	Equation/Reaction	Description
1	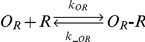	Reversible binding of ScbR to operator site O_R_
2	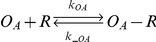	Reversible binding of ScbR to operator site O_A_
3	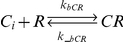	Reversible binding of SCB1 to ScbR
4	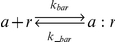	Reversible binding of full-length transcripts *a* and *r*

**Table 2:** Rates and Mass-action Equations for ScbA-ScbR model.

Equation No.	Equation	Description
5	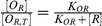	Equilibrium relationship between number of unoccupied O_R_ sites to total number of O_R,T_ sites
6	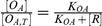	Equilibrium relationship between number of unoccupied O_A_ sites to total number of O_A,T_ sites
7	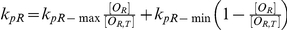	RNAP binding rate at promoter pR
8	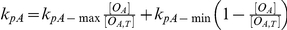	RNAP binding rate at promoter pA

In order to estimate the transcription rate from promoters pA and pR, the effect of convergent transcription is taken into consideration. The RNAP binding rate at promoters pA and pR is considered proportional to the concentration of de-repressed operator sites (unbound by R) and is given by Equations 7 and 8, where 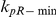 and 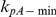
are the basal transcription rates under repressor-bound conditions, and 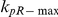
and 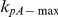 are the transcription rates under de-repressed (unbound) conditions from promoters pR and pA, respectively. The RNAP binding rate at pR and pA (respectively denoted by 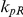 and 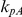
in Equations 7 and 8) is then the combined contribution of basal (repressed) and de-repressed rates. This is also the transcription rate of full-length *r* and *a* RNA in the absence of TI effects.

**Figure 3:**
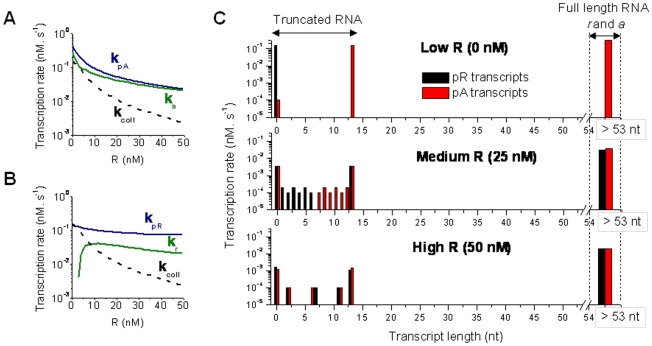
Transcriptional Interference within *scbA-scbR* locus. (**A–B**) Transcription rates *k_a_* and *k_r_* from promoters pA and pR respectively in presence of TI, the RNAP firing rates 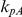 and 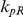
from promoters pA and pR respectively in absence of TI, and the net rate of RNAP collisions 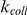 due to sitting duck collisions and collision between elongating RNAP, shown for different concentrations of repressor ScbR. (**C**) Transcription rate of different sizes of truncated RNA (<53 nt) and full-length RNA (*a* and *r*) from promoters pR and pA for different levels of repressor R.

The overall success and failure rate of pR and pA initiated transcription depends on the relative RNAP binding rates, *k_pR_* and *k_pA_* (Equation 7–8), the time taken to transition from a closed promoter complex to an elongation complex (τ) and the RNAP traveling time within the overlapping DNA. To determine the transcription rate of *a*, *r* for use in the ordinary differential equation (ODE) model (Table 2), we employed discrete simulation to calculate the formation rate of *a* and *r* RNA species over time. In the discrete simulation, RNAP binds to both pA and pR at time t = 0 and starts transcription after a τ = 2 second delay [44], which is was kept lower than the minimum RNAP binding time interval at promoters pA and pR (2.2 s and 4.8 s respectively) when concentration of R tends to zero. In the simulations, τ for pA and pR was kept the same. In absence of RNAP collision, thereafter, RNAP fires from pR and pA at rates *k_pR_* and *k_pA_*, respectively. Since both *k_pR_* and *k_pA_* are a function of R concentration (Equation 7–8), the simulation was carried out for different R concentrations (Fig. 3A–C). The velocity of an elongating RNAP is set at v_0_ = 50 bp/s [45]. RNAP is assumed to move along the DNA at a time step of 1/v_0_ for every base. Movement of RNAP was tracked along both strands of DNA within the overlapping region. The model does not consider potential RNAP pausing for a short stretch of overlapping DNA such as 53 bp. The parameters used for the simulation are summarized in Table 3.

**Table 3:** Parameter values and their range for which bistability is observed.

Parameter	Description	Estimated value for bistability	Range of bistability		Remarks/Reference	Units
			Min. evaluated	Max. evaluated		
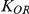	Equilibrium binding constant of ScbR to 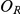	8.82	0.44	37.92	[69], [70]	nM
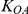	Equilibrium Binding constant of ScbR to 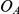	2.68	0.12	4.92	[69], [70]	nM
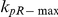	Transcription from P_R_ in de-repressed state	1.5×10^−1^	7.5×10^−3^	3.0×10^−1^	[65]	s^−1^
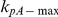	Transcription from P_A_ in de-repressed state	4.5×10^−1^	2.25×10^−2^	9.0×10^−1^	[65]	s^−1^
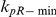	Transcription from P_R_ in repressed state	1.0×10^−3^	5.0×10^−5^	3.0×10^−3^	[65]	s^−1^
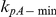	Transcription from P_A_ in repressed state	8.0×10^−4^	4.0×10^−5^	2.8×10^−3^	[65]	s^−1^
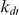	Degradation of full-length *r* RNA	7.0×10^−3^	3.5×10^−4^	1.4×10^−2^	[72]	s^−1^
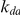	Degradation of full-length *a* RNA	8.1×10^−4^	4.1×10^−4^	1.6×10^−2^	[72]	s^−1^
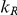	ScbR protein translation	3.6×10^−1^	9.0×10^−2^	3.8×10^−1^	[68], [69]	s^−1^
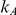	ScbA protein translation	6.6×10^−2^	1.0×10^−2^	1.2×10^−1^	[68], [69]	s^−1^
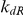	ScbR protein degradation	4.0×10^−3^	1.0×10^−3^	8.0×10^−3^	[69]	s^−1^
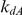	ScbA protein degradation	1.8×10^−3^	8.0×10^−4^	2.0×10^−3^	[69]	s^−1^
*µ*	growth rate	6.0×10^−5^	6.0×10^−5^	8.0×10^−5^	[71]	s^−1^
*k_C_*	SCB1 synthesis	7.4×10^−1^	7.4×10^−2^	37	[66]	s^−1^
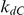	SCB1 degradation	6.7×10^−5^	6.7×10^−6^	6.7×10^−3^	Max. half life 1 hr.	s^−1^
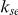	SCB1 secretion	8.3×10^−2^	8.3×10^−2^	4.2	[67]	s^−1^
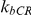	Binding of ScbR and SCB1 to form SCB1-ScbR complex	8.3×10^−2^	4.2×10^−3^	2.53×10^−1^	[70]	nM^−1^ s^−1^
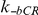	Unbinding of SCB1-ScbR complex	1.7×10^2^	8.5	1.95×10^2^	[70]	s^−1^
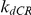	SCB1-ScbR degradation	3.1×10^−3^	3.1×10^−3^	6.8×10^−2^	[70]	s^−1^
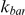	Binding rate constant of full-length (*a*, *r*) to form hybrid RNA complexes *a:r*	1.0×10^−3^	6.5×10^−4^	1.6×10^−1^	[48]	nM^−1^s^−1^
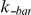	Unbinding of *ar* complex	1.0×10^−2^	0	2.0×10^−1^	[48]	s^−1^
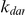	RNA hybrid complex *ar* degradation	1×10^−2^	0	1.24	[48]	s^−1^

The resulting apparent transcription rates of *a*, *r* to be used in the ODE model (Equation 9-10) are shown in Figure 3A-B. In absence of TI, the transcription rate of *r* RNA (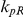) increased about two fold under derepressed conditions (low R concentration) compared to repressed state (high R concentration) (Fig. 3B). The presence of RNAP collision altered the dynamics of *r* transcription. Instead of having a modest increase at low R concentrations, the transcription rate of *r* (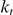) decreases to nearly zero (Fig. 3B). The vast majority of RNAP binding to pR are predicted to be knocked off by colliding RNAP from pA because of higher RNAP firing frequency from pA (3-fold higher than pR). At such a low level of R, full length *r* is reduced drastically. As expected, the RNAP collision rate increases with the increasing RNAP binding rates when R concentration decreases (Fig. 3A-B), consequently rate of production of very short truncated RNA (<13 nt) increases (Fig. 3C). The size distribution of the truncated RNA varies over the range of transcription initiation rates (Fig. 3C), however, these short truncated RNA lack secondary structure and thus are not considered to have antisense effects. At high R concentration when pA and pR strengths are low and comparable, both transcription initiation and collision rates are low, thus the truncated RNA generation rate is low, with a larger fraction located near either promoter. At moderate and high concentrations of R, *a* and *r* transcription rates are comparable. This is consistent with previously reported experimental data [26]. Theoretical and experimental analysis in bacteriophage 186 have shown that typically transcriptional interference in convergent promoter systems with shorter overlapping DNA is mainly due to sitting duck collisions [35], [40], [46]. Our simulation data also indicates that sitting duck is a major contributor to transcriptional interference in the *scbA-scbR* system.

The antisense interactions between *a* and *r* is modeled as second-order reaction (Equations 9–10 and 15). The value of rate constant 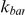 (1×10^−3^ nM^−1^ s^−1^) used is in the same range as reported in literature [47], [48]. The dissociation of the duplex RNA complex is assumed to follow first-order kinetics with a rate constant of 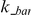
(Equations 9–10 and 15) [42], [47], [48]. The sense-antisense RNA duplex complex is assumed to be either degraded or otherwise unavailable for translation (Fig. 1B) [12], [42], [49]. The translation of ScbR and ScbA proteins is assumed to occur only from full-length transcripts, *r* and *a*, as shown by the first term in Equations 11 and 12. The binding of SCB1 to ScbR follows second-order kinetics (Equation 11 and 13–14) [28]. The dissociation of the CR complex is assumed to follow first-order kinetics, denoted by the term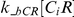 in Equations 11 and 13–14 [28]. The balance of SCB1-ScbR (*C_i_R*) complex is given in Equations 14. The rate of transport of SCB1 (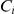) into or out of the cell is assumed proportional to the concentration difference of SCB1 across the cell membrane, as denoted by the *k_se_*(*C_i_* - *C_e_*) term [28]. Degradation of 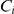 is considered to be a first-order process (Equation 13) [28].

### Convergent transcription confers a bistable *scbA/scbR* genetic switch

**Figure 4:**
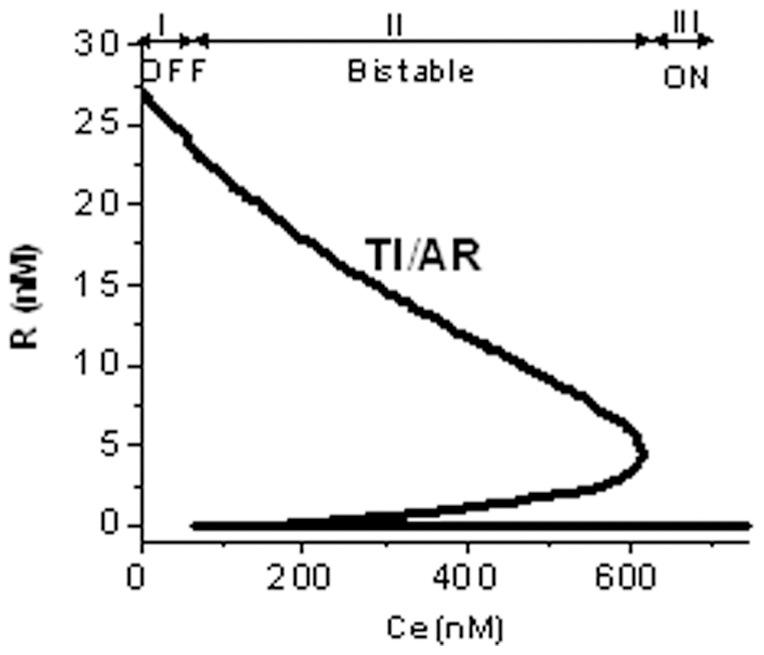
Bistable steady state response of ScbA/ScbR system to extracellular SCB1 in presence of convergent transcription. Steady state response of ScbR to constant extracellular SCB1 concentration. Parameters used for simulation are listed as nominal case in Table 3. Bistability is predicted in the case of convergent transcription. Region I and II indicate the monostable region of bistable curve corresponding to OFF and ON states respectively. Region III indicates the bistable region. The state of the system depends on its history. System originating from OFF state continues to be OFF in the bistable region, while system originating from ON state, stays ON in the bistable region. The system requires a higher concentration of SCB1 to turn ON than to turn back OFF.

Steady-state behavior of the *scbA-scbR* gene network was evaluated by numerically solving Equations 5–15, while keeping the extracellular SCB1 concentration (C_e_) constant. A characteristic bistable response of ScbR to extracellular SCB1 is predicted, as shown in Fig. 4. At low concentrations of SCB1 (C_e_<66 nM), the system demonstrates a single high R (OFF) state, while at high SCB1 (C_e_ >616 nM), the R concentration is low. Since R is a direct regulator of the downstream *cpk* gene cluster, high expression levels of R indicates an OFF state, whereas low levels of R indicates an ON state for *cpk* gene cluster. Three regions of steady states are seen. Region I (C_e_<66 nM) corresponds to a stable OFF steady state with no antibiotic production. Region II corresponds to an ON state (C_e_>616 nM) of antibiotic production. The intermediate region III has two stable steady states (corresponding to either ON or OFF), in addition to one unstable (unobservable) state. Depending on the history of the system, i.e. whether the system was originally in an ON or OFF state, the trajectory of the system differs: When the system is in the initial OFF state, it continues to be in OFF state until C_e_ reaches 616 nM, at which point the system switches to ON (Fig. 4). On the other hand, starting from an ON state, as C_e_ is decreased, the switch does not occur until a value of 66 nM is reached. Thus, in region III the system is bistable, showing alternative steady states, and therefore relatively protected against small spurious fluctuations in SCB1 concentration. Table 3 presents the range of parameters where bistability was observed.

### Contribution of Transcriptional interference and antisense regulation to bistability

**Figure 5:**
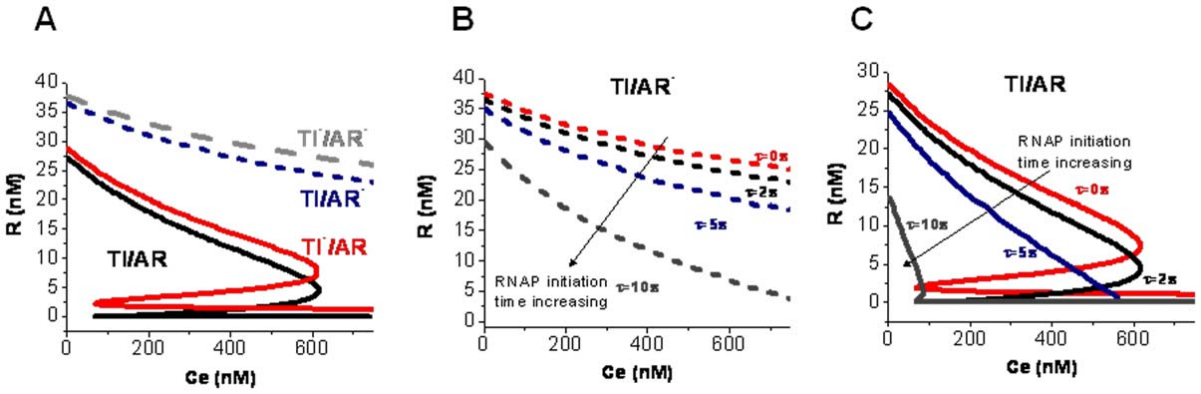
Relative contribution of RNAP collision and antisense regulation effects to bistable switch response. (**A**) Bistability is lost in the absence of TI and antisense RNA interaction effects (TI^−^/AR^−^). In presence of only TI effects (TI/AR^−^), the bistable behavior is lost, system ceases to behave as a switch. Bistability is restored when only antisense RNA interaction between full-length RNA is considered in absence of RNAP collision (TI^−^/AR). The system exerts tighter regulation on ScbR expression in presence of convergent transcription (TI/AR). (**B**) Steady state expression level of repressor R to extracellular SCB1 in presence of TI effect only for different RNAP initiation time (τ) at promoter pA and pR. (**C**) Steady state expression level of repressor R to extracellular SCB1 in presence of both TI and AR effects for different RNAP initiation time (τ) at promoter pA and pR.

We evaluate the steady state behavior under the conditions of no Transcriptional interference (TI^−^) and/or no antisense regulation (AR^−^) (Fig. 5A). TI effect is eliminated from the model by assigning rates of production of full-length transcripts 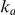 and 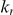
equal to the RNAP binding rate from pA and pR, i.e. 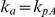 and 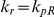. The AR effect is removed by setting the parameters 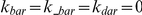. Without AR and TI, the system loses bistability (Fig. 5A, TI^−^/AR^−^ case). A broad scan of the parameter space did not yield a bistable response for any selection of parameters (Table 3). Removing only antisense regulation but not TI, by setting the parameters 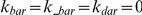 eliminated the bistable behavior (Fig. 5A, TI/AR^−^ case). With antisense regulation alone without TI effect (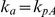 and 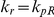) the system could still demonstrate bistability (Fig. 5A, TI^−^/AR case). Antisense regulation is thus critical for the bistable response in this system. Interestingly, when both TI and AR effects are considered, the system requires a lower value of R to transition into the ON state compared to the case of AR alone. The combined effect of TI and AR imposes a slightly higher suppression of R at the OFF state.

A factor affecting TI is the time taken by a closed promoter complex to transition into an elongation complex, or the RNAP initiation time τ. The effect of τ on bistable behavior is assessed by varying its value between 0 s (very fast) to 10 s (very slow). In the course of such assessment we incorporate the effect of τ on the transcription initiation rate, as a long duration of RNAP occupation at a promoter will decrease the rate of transcription initiation at high promoter strengths (Fig. S1). RNAP is assumed not to bind at a promoter, till the promoter is cleared. The steady state response of R to C_e_ in presence of TI effect alone showed a ramping-down behavior (Fig. 5B). For high values of τ (Fig. 5B) the R concentration moves from a OFF to an ON state, albeit in a ramping fashion. For low values of τ, the system stays at an OFF state even at high levels of C_e_. However, bistability is not seen in the case of TI alone (Fig. 5B). When both TI and antisense effects (TI/AR) are present, as τ increases, the bistable region decreases and at high values of τ bistability is lost. At high τ, the transcription rates of full-length *a* and *r* RNA decrease (Fig. S1), resulting in weakening of antisense effects. Thus, decreasing the relative contribution of antisense effect to TI effect causes loss of bistability, indicating that the non-linear effect offered by antisense interactions between the full-length RNA is essential for bistability (Fig. 5C).

### Sensitivity of the bistable response

**Figure 6:**
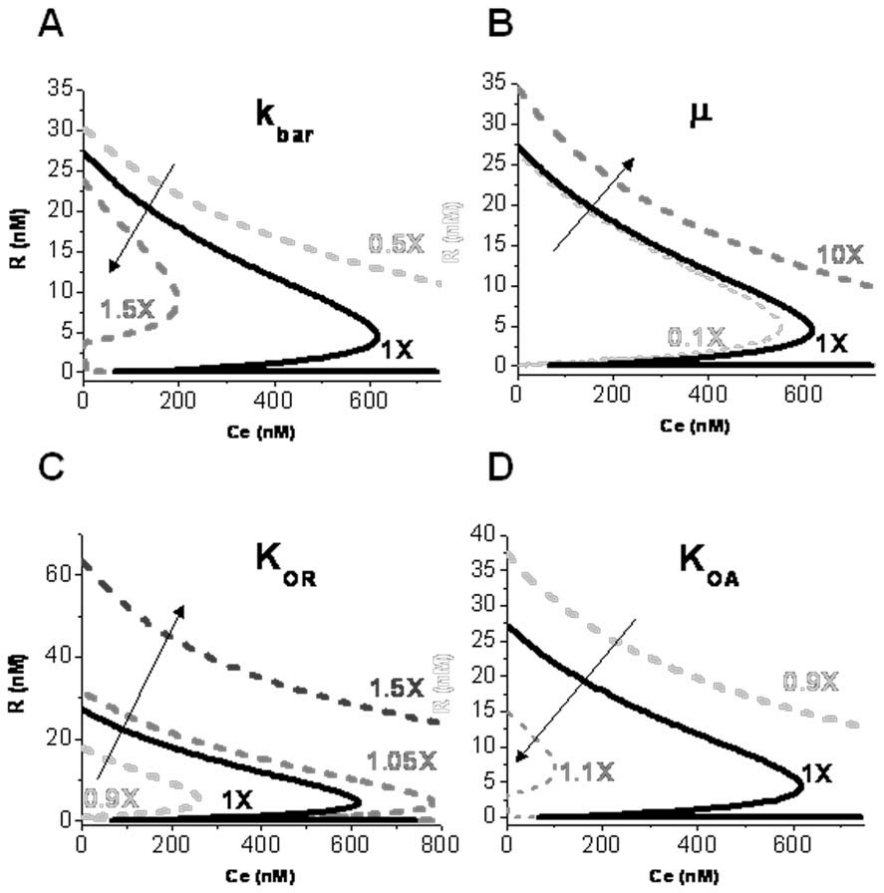
Effect of single-parameter perturbation on steady state response of ScbA-ScbR system to constant extracellular SCB1. Results show the effect of varying one (indicated) parameter while keeping the rest constant at the nominal values listed in Table 3. The solid black line (1X) in each plot corresponds to the nominal parameter values. Effect of changing parameters: (**A**) Sense: antisense RNA interaction rate constant, (**B**) growth rate and (**C–D**) equilibrium rate constant corresponding to binding of repressor R to operator sites O_R_ (C) and O_A_ (D) on the steady state response of ScbR to extracellular SCB1.

We evaluated how the steady state behavior of ScbA/R system is affected by a number of parameters critical to the observed bistability. The key outcome of antisense regulation is the decrease in *a* and *r*. We evaluated the effect of antisense interaction by varying the value of 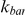, while keeping the values of the remaining parameters constant. As 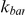
is decreased, the bistable region diminishes. Eventually, the bistable response becomes a ramping response (Fig. 6A) approaching the TI only case as described in Fig. 5B. Increasing 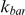 caused the system to shift from reversible bistable behavior to one that is irreversible with the bistable region extending to the zero concentration of C_e_. A system that originates from an ON state (low ScbR) will remain ON with decreasing C_e_. This is explained by the fact that high rates of binding of sense and antisense RNA (high 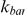) will sequester free *r* RNA. As a result the system will not be able to produce enough repressor R to allow the system to return back to the OFF condition.

In *S. coelicolor* cultures, SCB1 and antibiotic production commence during the transition phase from a rapid growth to a slower growth [26]. We evaluated the effect of growth rate on bistability. Increasing the growth rate causes the bistable curve of the response of R to SCB1 to shift to the right; however, the bistable region shrinks and ultimately vanishes at very high values of the specific growth rate (Fig. 6B). At 10 fold decreased growth rate than the nominal values used in Table 3, the system demonstrates irreversible bistability. This implies, that at slower growth rates such as in stationary phase, once the switch is ON, the system continues to stay ON, the cells will continue the production of antibiotics and/or secondary metabolites, until the growth rate increases again.

### Effect of ScbR repression on promoters pA and pR

The strength of promoters pA and pR is critical to the bistability. Since the repressor R is a direct effector of the promoter strength, we next evaluated the effect of ScbR repression on promoters pR and pA. The repressive effect of R on promoters pA and pR is characterized by the equilibrium binding rate constant of R at the operator sites O_R_ and O_A_, 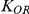 and 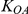, respectively. Higher 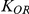 and 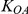 implies lower binding affinity of R towards operators O_R_ and O_A_, respectively, hence correspondingly a lower repressive effect on promoters pR and pA. The effect of changing 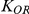 on the response of R to SCB1 is shown in Fig. 6C. In this case, decreasing 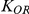 results in an irreversible bistable switch, implying tighter regulation of pR by R prevents the system from turning OFF. Also, the steady state level of R in the OFF state is lower than the nominal case. The tighter repression of R causes a lower expression level of R: once the system is ON, the system is never able to switch OFF, as the excess SCB1 sequesters the free R. Increasing 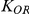, i.e. decreased repression, causes the bistable curve to shift to the right along with widening of the bistable region, implying that a higher SCB1 concentration is required to induce the system. Changing the rate constant 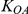 shows an effect opposite to that of 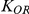 (Fig. 6D). When increasing 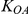, thus decreasing binding affinity of R to O_A_, the system begins to demonstrate irreversible bistability. On the other hand, with decreasing 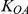, the bistable region expands considerably, such that the system turns ON only at much higher SCB1 concentration.

## Discussion

Cellular decisions are mediated through genetic switches which arise from interactions between simple biological molecules. Robust genetic switches often demonstrate bistability, which implies that the system exists only in two discrete states, i.e. cells either exist in an ON state or an OFF state [50]. Several key physiological decisions such as the transition between lysogeny and lytic state in bacteriophage λ [51], determining competence in *Bacillus subtilis* [52], or between differentiation and self-renewal in stem-cells [53], [54], have been shown to be characterized by bistable behavior. Bistable systems usually demonstrate hysteresis, making them less susceptible to fluctuating noise around the decision point, as the threshold required for the system to switch from OFF to ON is different from that going back from ON to OFF.

*S. coelicolor* switches from a vegetative growth state (antibiotic production OFF) to a stationary state (antibiotic production ON) [26], [28]. The decision to switch from OFF to ON is a critical one, as secondary metabolites such as antibiotics can be toxic to the producers themselves. The switch from the vegetative to the antibiotic-producing state is triggered when the concentration of extracellular SCB1 reaches a critical threshold. Previously, we have shown that formation of a hypothetical ScbA-ScbR protein complex acting as a positive regulator of transcription from pA [28], would be required for bistable switching behavior. Removal of the ScbA-ScbR complex from the model resulted in loss of the switch response as demonstrated by the TI^−^/AR^−^ case in the current analysis (Fig. 5A).

In this work we have shown that convergent transcription in the *scbA-scbR* locus can restore the bistable switch behavior in the absence of ScbA-ScbR protein complex. Interestingly, we found that convergent transcription gave rise to bistable behavior over a wider parameter range (Table 3) compared to the earlier model that depended on an ScbA-ScbR protein complex [28]. This robustness is an implicit argument in favor of the convergent transcription mechanism [55], [56]. The bistable behavior in the ScbA/ScbR system appears to be robust as it is relatively insensitive to the value of the parameters used. Varying the value of the parameter both individually (Fig. 6, Fig. S2 and S3) and in combination with other parameters we examined the maximum range of system bistability for each parameter (Table 3). Parameter space search showed that bistable behavior is retained for at least an order of magnitude range for majority of parameters.

Convergent transcription gives rise to two mechanisms of gene regulation: transcriptional interference and antisense interaction. Transcriptional interference has been shown to play a regulatory role in the expression from pR-pL promoter pair in bacteriophage 186 [35], P_Q_-P_X_ promoter pair controlling conjugation of pCF10 plasmid in *Enterococcus faecalis* [11], S-box antisense RNA repression of *ubi-mccBA* mRNA of *C. acetobutylicum* [57] and in cell fate control between diploid and haploid states in *IME4* locus of *Saccharomyces cerevisiae* [58]. In ScbA-ScbR system, a single repressor ScbR regulates expression from both promoters. At high levels of repressor ScbR, expression from promoter pR and pA are comparable (Fig. 3A–C). At low free R conditions, transcription rate from the pA promoter is higher than from the pR promoter. pA thus functions as the dominant promoter and its transcription suppresses transcription from pR mainly via sitting duck collisions and occlusion of pR promoter. The resultant transcriptional interference has two effects on transcription: first, decreasing the expression of full-length transcripts from both promoters; second, the effect of TI is more severe for transcription from the weaker promoter, thus resulting in the amplification of the difference of the levels of the transcripts from the two promoters.

The probability of RNAP collision in the overlapping region, thus the effect of TI, is affected by a number of factors. A longer overlapping region increases the TI effect due to higher probability of RNAP collision [11], [35], [36]. For a given length of overlapping sequence RNAP collision frequency is increased in the presence of pause sites within the overlapping region as reported for the case of PR-PRE promoter pair in bacteriophage λ [35], [36]. Similarly, increasing the time interval of RNAP binding to promoter to transcriptional initiation also increases sitting duck collisions [35]. In the *scbA-scbR* system it results in further decrease in the production of full-length RNA (Fig. S1A–B).

Antisense interaction is the second layer of regulation offered by convergent transcription. Full-length transcripts have complementary counterparts from the opposing promoters which may elicit antisense interactions. The resulting hybrid RNA complex are subjected to degradation or rendered untranslatable [42], [59]. In this system we assume RNA interaction tends to sequester full-length transcripts and prevent their translation. The effect is more severe for the less abundant RNA, as the depletion effect is more pronounced. Similar role of antisense RNA in down regulating synthesis of proteins from *cis-*encoded genes has been reported in other systems, including *Sok* mRNA of plasmid R1 in *E.coli* [49], antisense RNA *alr1690-furA* regulating expression of transcriptional repressor FurA in cyanobacterium *Anabena* sp. PCC 7120 [12] and other systems reviewed in [60]. The repressive effect of antisense transcripts has also been experimentally shown between the *prgQ* and *prgX* transcripts of plasmid pCF10 in *Enterococcus faecalis* [11], 1200 nt *AmgR* RNA encoded convergent to *mgtCBR* operon in *S*. *enterica* [13], 108 nt RNAI RNA controlling copy number of plasmid ColEI [61], 69 nt Sar RNA of bacteriophage 22 repressing Ant protein [62] and 77 nt OOP RNA of bacteriophage λ repressing CII protein [63].

Recent work has led to the discovery of *cis* aRNA in *Streptomyces coelicolor* [14], [15], [16]. Nearly 3600 *cis* non-coding RNA have been predicted, some of these that were experimentally validated show differential expression under certain growth conditions [14]. For example, over expression of chromosomal *cis* non-coding RNA *cnc2198.1* found antisense to glutamine synthetase I has been shown to result in decrease in protein expression, growth and production of antibiotics in *S.coelicolor* [14]. Given the widespread presence of convergent transcription in both prokaryotic and eukaryotic organisms, it is highly plausible that the arrangement of gene pairs in such organization confers some biological regulatory function. It has been reported that 1947 such convergent promoter pairs are present in the mouse genome and transcriptome analysis provides evidence that a significant fraction of these have asymmetrical transcriptional regulation [64].

The *Streptomyces coelicolor* genome consists of 1429 pairs of divergently transcribed genes, however, transcript start sites have been determined for only a couple of cases (http://streptomyces.org.uk/). Based on known open reading frames, at least 80 gene pairs are arranged in convergent orientation, although the actual extent of transcript overlap is not known. The asymmetry in transcription rates from genes with convergent promoters could change upon induction (de-repression), analogous to the ScbA-ScbR system discussed in this work, a phenomenon that could operate in many of the convergent promoter pairs present in *S. coelicolor*. It is possible that mechanisms of transcriptional interference and antisense regulation operate in these convergent promoter systems and play regulatory roles in gene expression. Despite the structural simplicity, convergent gene-pairs may harbor some regulatory complexity yet to be fully investigated and exploited.

## Materials and Methods

### Steady state and dynamic analysis of mathematical model

Numerical solutions to the ordinary differential equations were solved using the stiff differential equations solvers ode23s in Matlab®. The steady states for the equations were computed in Mathematica. The stability of solutions obtained was characterized by Eigenvalues of the Jacobian. The complete set of kinetic parameters involved in the above model is listed in Table 3. The range of values for each parameter in Table 3 was obtained from the literature [65], [66], [67], [68], [69], [70], [71], [72]. The parameter range was explored to determine the capability of such a system to show the desired system dynamics. *In silico* structures of RNA were generated using Sfold software [73].

## Supporting Information

Figure S1 **Effect of different RNAP initiation time (τ) on the resultant transcriptional interference.** At high values of τ (e.g. τ = 5 s and 10 s), the time required for RNAP initiation at the promoter is longer than the RNAP binding interval at low concentrations of repressor (i.e. RNAP binding time intervals of 2.2 s and 4.8 s at promoters pA and pR respectively for [R] = 0 nM). In such as case, RNAP is assumed not to bind at a promoter, till the promoter is cleared. This is implemented in the simulations by aborting the n^th^ round of RNAP binding at a promoter and resuming it at n+1^th^ round. (**A**) Rate of transcription of full-length RNA (*k*_r_ and *k_a_*) and (**B**) rate of transcription of full-length RNA normalized to RNAP binding rates (*k_r_*/k_pR_ and *k_a_*/k_pA_), for different RNAP initiation time (τ) at promoters pR and pA, at different concentrations of repressor ScbR.
(TIF)

Figure S2 **Effect of single-parameter perturbation for parameters describing transcription and translation on the steady state response of ScbA-ScbR system to constant extracellular SCB1.** Results show the effect of varying one (indicated) parameter while keeping the rest constant at the nominal values listed in Table 3. The solid black line (1X) in each plot corresponds to the nominal parameter values described in Table 3. The parameter being varied include, transcription rate constants: (A) *k_pR-max_* (B) *k_pA-max_* (C) *k_pR-min_* (D) *k_pA-min_*, and translation rate constants: (E) *k_R_* (F) *k_A_*.
(TIF)

Figure S3 **Effect of single-parameter perturbation on steady state response of ScbA-ScbR system to constant extracellular SCB1.** Results show the effect of varying one (indicated) parameter while keeping the rest constant at the nominal values listed in Table 3. The solid black line (1X) in each plot corresponds to the nominal parameter values described in Table 3. The parameter being varied include degradation rates: (A) *k_dr_* (B) *k_da_* (C) *k_dR_* (D) *k_dA_* (E) *k_dC_* (F) *k_dCR_* (G) *k_dar_*, (H) SCB1 secretion rate: *k_se_*, (I) SCB1-ScbR binding rate constant, *k_bCR_*, Unbinding rate constants: (J) *k__bCR_* (K) *k__bar_* and (L) SCB1 production rate, *k_C_*.
(TIF)
