# Supplementary figures and images for: Convergent Transcription in the Butyrolactone Regulon in Streptomyces coelicolor Confers a Bistable Genetic Switch for Antibiotic Biosynthesis
Source: PLoS One. 2011 Jul 12;6(7):e21974. doi: 10.1371/journal.pone.0021974 (PMC3134472; doi:10.1371/journal.pone.0021974)

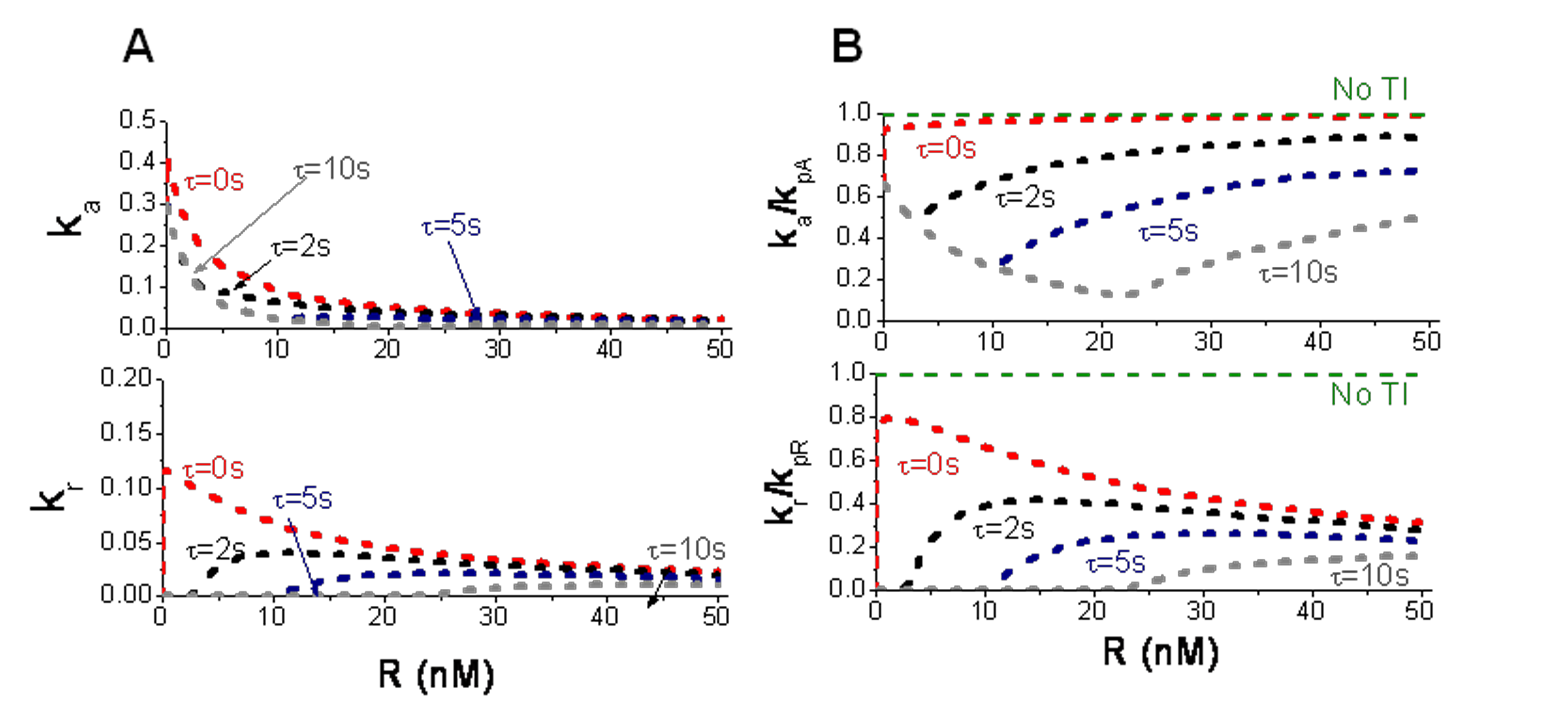

Supplement: Figure S1 — Effect of different RNAP initiation time (τ) on the resultant transcriptional interference. At high values of τ (e.g. τ = 5 s and 10 s), the time required for RNAP initiation at the promoter is longer than the RNAP binding interval at low concentrations of repressor (i.e. RNAP binding time intervals of 2.2 s and 4.8 s at promoters pA and pR respectively for [R] = 0 nM). In such as case, RNAP is assumed not to bind at a promoter, till the promoter is cleared. This is implemented in the simulations by aborting the nth round of RNAP binding at a promoter and resuming it at n+1th round. (A) Rate of transcription of full-length RNA (k r and ka) and (B) rate of transcription of full-length RNA normalized to RNAP binding rates (kr/kpR and ka/kpA), for different RNAP initiation time (τ) at promoters pR and pA, at different concentrations of repressor ScbR. (TIF) [file pone.0021974.s001.tif]

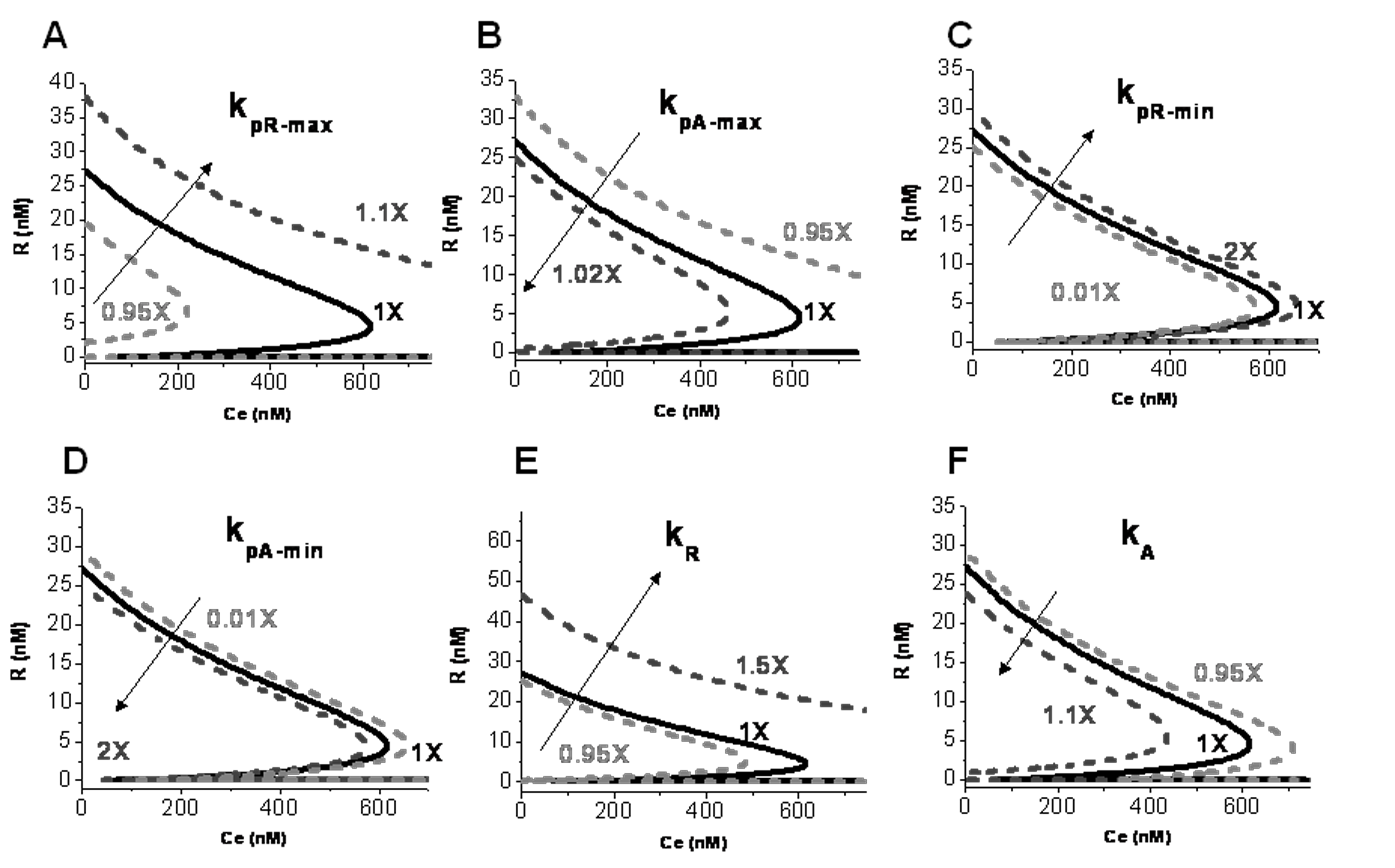

Supplement: Figure S2 — Effect of single-parameter perturbation for parameters describing transcription and translation on the steady state response of ScbA-ScbR system to constant extracellular SCB1. Results show the effect of varying one (indicated) parameter while keeping the rest constant at the nominal values listed in Table 3. The solid black line (1X) in each plot corresponds to the nominal parameter values described in Table 3. The parameter being varied include, transcription rate constants: (A) kpR-max (B) kpA-max (C) kpR-min (D) kpA-min, and translation rate constants: (E) kR (F) kA. (TIF) [file pone.0021974.s002.tif]

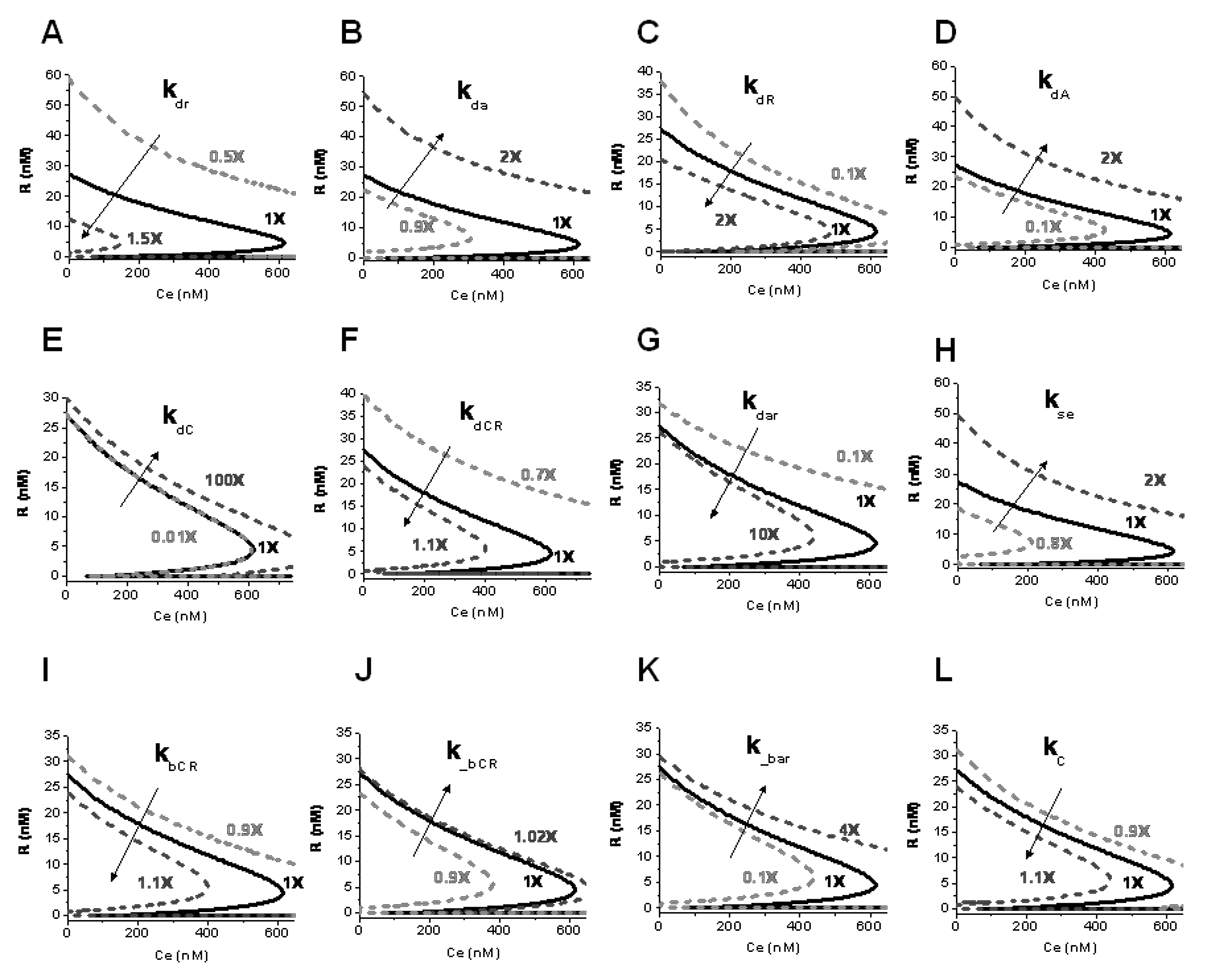

Supplement: Figure S3 — Effect of single-parameter perturbation on steady state response of ScbA-ScbR system to constant extracellular SCB1. Results show the effect of varying one (indicated) parameter while keeping the rest constant at the nominal values listed in Table 3. The solid black line (1X) in each plot corresponds to the nominal parameter values described in Table 3. The parameter being varied include degradation rates: (A) kdr (B) kda (C) kdR (D) kdA (E) kdC (F) kdCR (G) kdar, (H) SCB1 secretion rate: kse, (I) SCB1-ScbR binding rate constant, kbCR, Unbinding rate constants: (J) k_bCR (K) k_bar and (L) SCB1 production rate, kC. (TIF) [file pone.0021974.s003.tif]
